# Investigation of 9000 hours multi-stress aging effects on High-Temperature Vulcanized Silicone Rubber with silica (nano/micro) filler hybrid composite insulator

**DOI:** 10.1371/journal.pone.0253372

**Published:** 2021-07-28

**Authors:** Arooj Rashid, Jawad Saleem, Muhammad Amin, Sahibzada Muhammad Ali, Aftab Ahmad Khan, Muhammad Bilal Qureshi, Sara Ali, Darren Dancey, Raheel Nawaz

**Affiliations:** 1 Electrical and Computer Engineering Department, COMSATS University, Abbottabad, KPK, Pakistan; 2 Ghulam Ishaq Institute of Engineering Sciences, Swabi, KPK, Pakistan; 3 School of Mechanical and Manufacturing Engineering (SMME), National University of Sciences and Technology, Islamabad, Pakistan; 4 Manchester Metropolitan University, Manchester, United Kingdom; Changchun Institute of Applied Chemistry, Chinese Academy of Sciences, CHINA

## Abstract

Degradation in the polymeric insulators is caused due to the environmental stresses. The main aim of this paper is to explore the improved aging characteristics of hybrid samples by adding nano/micro silica in High Temperature Vulcanized Silicone Rubber (HTV-SiR) under long term accelerated aging conditions for 9000 hours. As HTV-SiR is unable to sustain environmental stresses for a long time, thus a long term accelerated aging behavior is an important phenomenon to be considered for field application. The aging characteristics of nano/micro filled HTV-SiR are analyzed by using techniques such as Scanning Electron Microscopy (SEM), Leakage Current (LC), Fourier Transform Infrared Microscopy (FTIR), Hydrophobicity Classification (HC), and breakdown strength for the aging time of 9000 hours. FTIR and leakage currents are measured after every cycle. All the co-filled samples revealed escalated aging characteristics as compared to the neat sample except the SN8 sample (8% nano-silica+20% micro-silica) after 9000 hours of aging. The highest loading of 6% and 8% nano-silica with 20% micro-silica do not contribute to the improved performance when compared with the neat and hybrid samples. However, from the critical experimental analysis, it is deduced that SN2 sample (2% nano-silica+20% micro-silica) is highly resistant to the long term accelerated aging conditions. SN2 has no cracks, lower loss percentages in the important FTIR absorption peaks, higher breakdown strength and superior HC after aging as compared to the unfilled and hybrid samples.

## 1 Introduction

Recently, the polymer insulators have gain more popularity over the ceramic insulators [1]. Among polymer insulators, High Temperature Vulcanized Silicone Rubber (HTV-SiR) is more acceptable due to the various improved characteristics like hydrophobicity recovery, lightweight, ease of handling, improved performance in polluted environment, and handling [2–4]. Due to improved characteristics, it is applicable for the overhead transmission line insulators for extra-high voltage and ultra-high voltage applications. HTV-SiR is an organic material having Si-O-Si as main chain with two methyl group (-CH3), that makes it a hydrophobic material with improved hydrophobicity characteristics. Despite the superior characteristics, the HTV-SiR is still under research due to degradation phenomenon when it is subjected to harsh weather/environmental conditions [5, 6]. Degradation phenomenon in the polymers exists due to the different stresses present in the environment, which is the main cause of aging in polymer insulators [7]. Aging phenomenon also effects the HTV-SiR. Some of the environmental stresses that cause the aging in polymer insulators are: rain, fog, temperature, ultra-violet (UV) rays and humidity [8, 9]. The multiple stresses must be taken into account along with the long term aging to evaluate the insulator [10]. The aging phenomenon usually causes the loss of hydrophobicity, increase in leakage current, lowers the electrical characteristic, mechanical, thermal, and reduced flashover voltage [2, 11, 12]. For the enhancement in the characteristics of HTV-SiR, usually a small portion of filler is added. These fillers are added according to the requirement for improved electrical, thermal, mechanical, and erosion/tracking characteristics. Some of the most commonly used inorganic filler for the improvement of characteristics are silica, alumina trihydrate, alumina, magnesium hydroxide, carbon black, and calcium carbonate [13–19]. Among all these fillers, silica is one of the widely used option. In [13], the micro and nano-silica is added in RTV-SiR. The results indicate that the sample with micro-sized silica was found to have improved tracking/erosion resistance as compared to the non-filled and nano-filled insulator. In [20, 21], alumina trihydrate and aluminum oxide has been used as filler and enhanced electrical, mechanical, thermal, and tracking/erosion was observed. A new concept of simultaneous addition of micro and nanoparticles emerges and requires further investigation. In [22], the authors added nano-silica and micro-alumina with varying percentage of nanoparticles which resulted in the improvement of the characteristics. However, the aging phenomenon effects different hybrid insulators differently and needs further investigation for the selection of insulator with improved aging characteristics.

This research work contributes towards the first time long-term aging characteristics of hybrids having high temperature vulcanized silicone rubber as base material for 9000 hours. The uniqueness of research lies in the preparation of hybrid samples and its long term accelerated aging analysis of 9000 hours in specific environmental conditions. This long term accelerated aging analysis of 9000 hours will ensure the reliability and authenticity of the hybrid samples, so they can be implemented in the power system. In this article, aging characteristics of hybrid sample are investigated and compared with the neat sample. The samples are prepared with the simultaneous addition of nano and micro filler in HTV-SiR. Following samples are prepared:

- 2% nano SiO_2_+20% micro SiO_2_ (SN2)
- 4% nano SiO_2_+20% micro SiO_2_ (SN4)
- 6% nano SiO_2_+20% micro SiO_2_ (SN6)
- 8% nano SiO_2_+20% micro SiO_2_ (SN8)
- Neat Sample (S0)

For evaluating the aging characteristics, a special plexiglass aging chamber is constructed. The samples are placed in this chamber for 9000 hours for further investigation. For analyzing the aging effects on these samples Hydrophobicity Classification (HC), Leakage Current (LC), breakdown strength and Fourier Transformed Infrared Spectroscopy (FTIR) is performed during the aging time of 9000 hours. Scanning Electron Microscopy (SEM) is performed before and after aging. The results are evaluated and then compared with the neat sample. After aging analysis, a superior sample is selected from all the samples that has improved aging characteristics.

The organization of the paper is as follow: Section 2 discusses the sample preparation, code and aging chamber details. Section 3 discusses the SEM analysis, HC, LC experimentation and analysis, breakdown strength, and FTIR analysis for the 9000 hours of aging respectively. Section 4 presents a detailed comparison of our research with the state-of-art research, and section 5 concludes the aging analysis of the hybrid samples.

## 2 Sample preparation, sample codes and aging chamber

**Fig 1:**
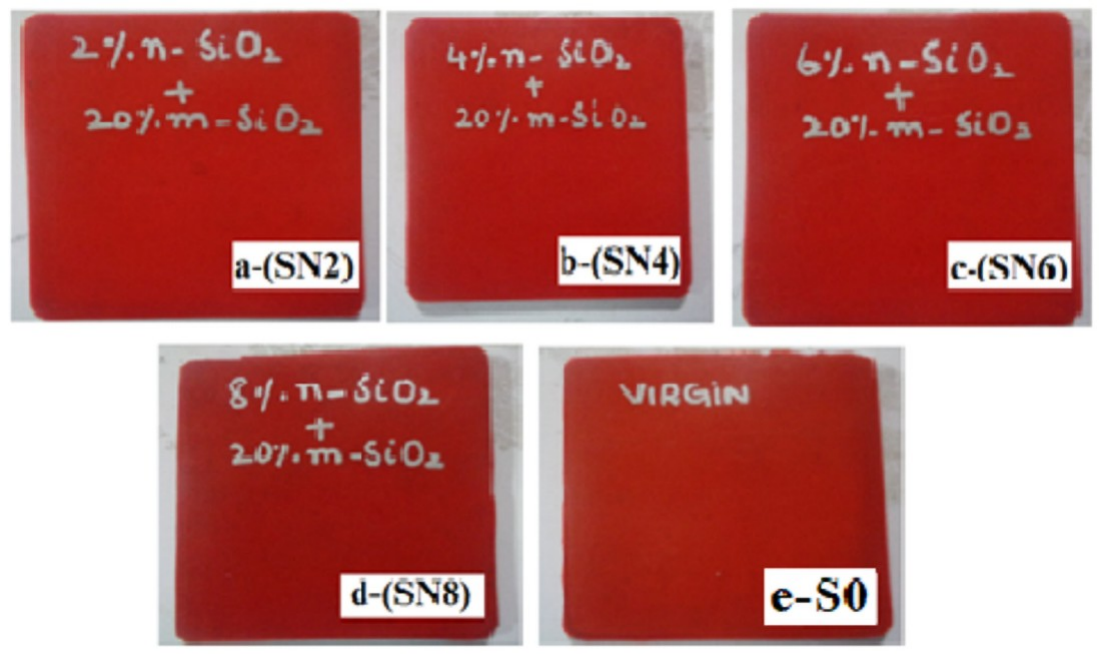
Original Samples, a- SN2, b-SN4, c-SN6, d-SN8 and e-S0.

Nano (size: 12 *n*m, density: 2.1 g/*m*^3^) and micro (size: 5 *μ*m, density: 2.52 g/*m*^3^) silica are acquired from Sigma Aldrich. All other chemicals used for the preparation process are of industrial grade. Methyl vinyl silicone rubber (colorless, molecular weight: 5800000, vinyl%:0.09, density: 2.54 g/*cm*^3^)is procured from DOW Corning, USA. Mixing and compounding method is used for the preparation of the sample. First of all, the fillers are suspended in 0.1 liter of ethanol. The mixture is stirred for 20 minutes. For preparation of the homogeneous mixture of filler in ethanol, ultrasonification is applied on the sample for 30 minutes. Silicone rubber is added in this solution under the process of sonication. For the removal of ethanol, the mixture is heated for an hour at 100°C. Later, it is placed in the mixture at 150 revolutions per minute (rpm) for 20 minutes at 150°C. This mixture is placed in pre-heated molds to get the required shape and size. The samples are cured at 170°C for 30 minutes. SiO_2_ nanoparticles with varying percentages of 2%, 4%, 6%, 8% are added in the HTV-SiR base material, keeping the percentage of micro silica constant (20%). A neat sample (S0) is also prepared along with the hybrid samples. The composition of all the samples is shown in Table 1. Fig 1 presents the photographs of all the samples.

**Table 1:** Sample code and their description.

Sample Code	Sample Composition
SN2	2% nano SiO2+20% micro SiO2 in HTV-SiR
SN4	4% nano SiO2+20% micro SiO2 in HTV-SiR
SN6	6% nano SiO2+20% micro SiO2 in HTV-SiR
SN8	8% nano SiO2+20% micro SiO2 in HTV-SiR
S0	Only HTV-SiR (neat)

**Fig 2:**
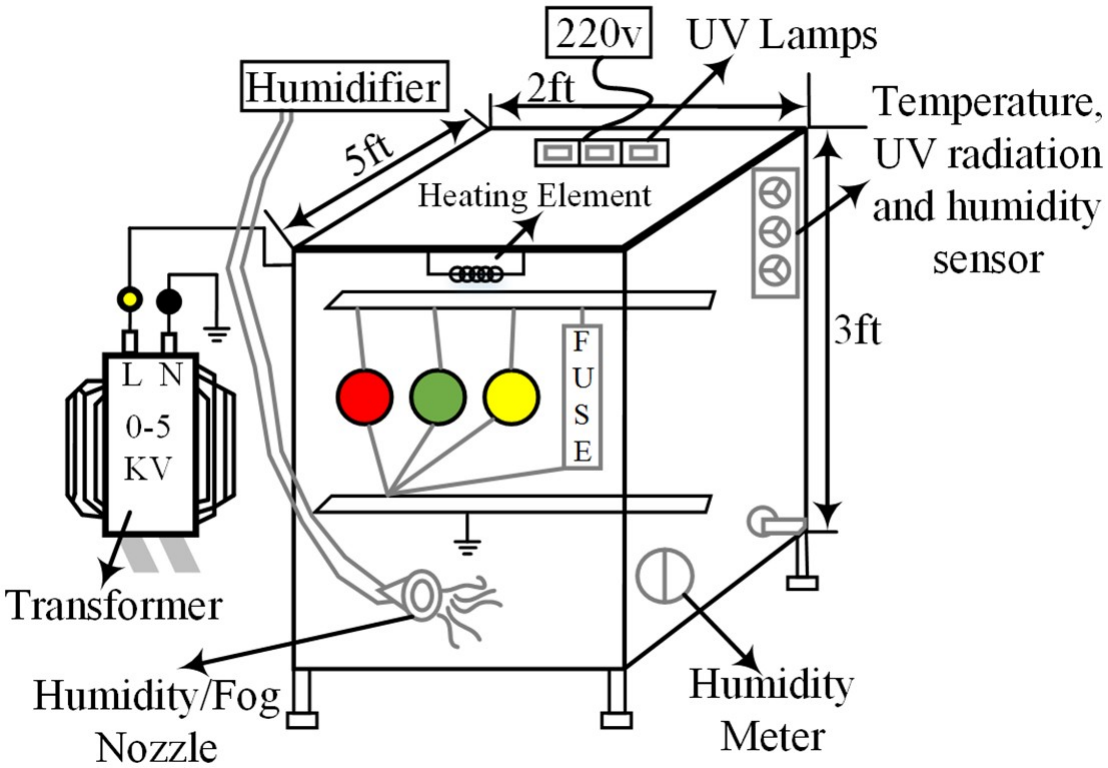
Aging chamber.

**Fig 3:**
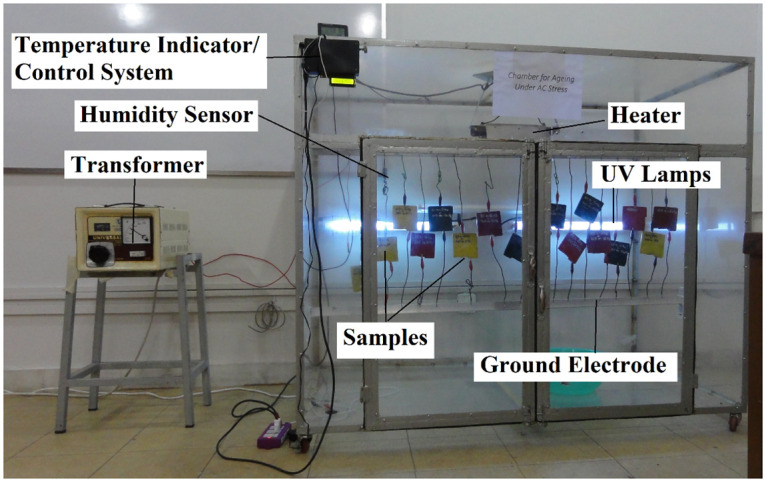
Real photograph of the aging chamber.

Depending upon the stresses applied on the sample, a chamber is fabricated to stimulate the aging environment. A 5*ft* × 3*ft* × 2*ft* plexiglass chamber is prepared as shown in Fig 2. The original picture of the aging chamber placed in the laboratory is shown in Fig 3. The samples are placed and stresses are applied simultaneously. A step up variac transformer of 220*V*/5*kV* is used to apply the electric stress. For the environmental stresses, UV radiations, heat, acid rain, fog, and humidity are applied. For UV radiation, three 20 watt UV lamps are installed in the chamber. 1000 Watt heater along with a controller is used to provide the desired amount of heat to the samples. For humidity, a humidity measuring element is used. The samples are placed in a chamber for 9000 hours. Summer and winter cycles are created and aged according to the conditions as shown in Table 2.

**Table 2:** Winter and summer cycle details.

Stresses	Summer Cycle	Winter Cycle
Applied Voltage	2.5 kV	2.5kV
Days	11	17
Temperature	47.3°C	35.2°C
UV radiation	10 hours per day	8 hours per day
Salt Fog(6000 *μ*S/cm)	0 times	4 times
Acid Rain (4.5 pH)	6 times	3 times
Humidity	77%	63%

## 3 Aging analysis techniques

### 3.1 Scanning Electron Microscopy (SEM) analysis

**Fig 4:**
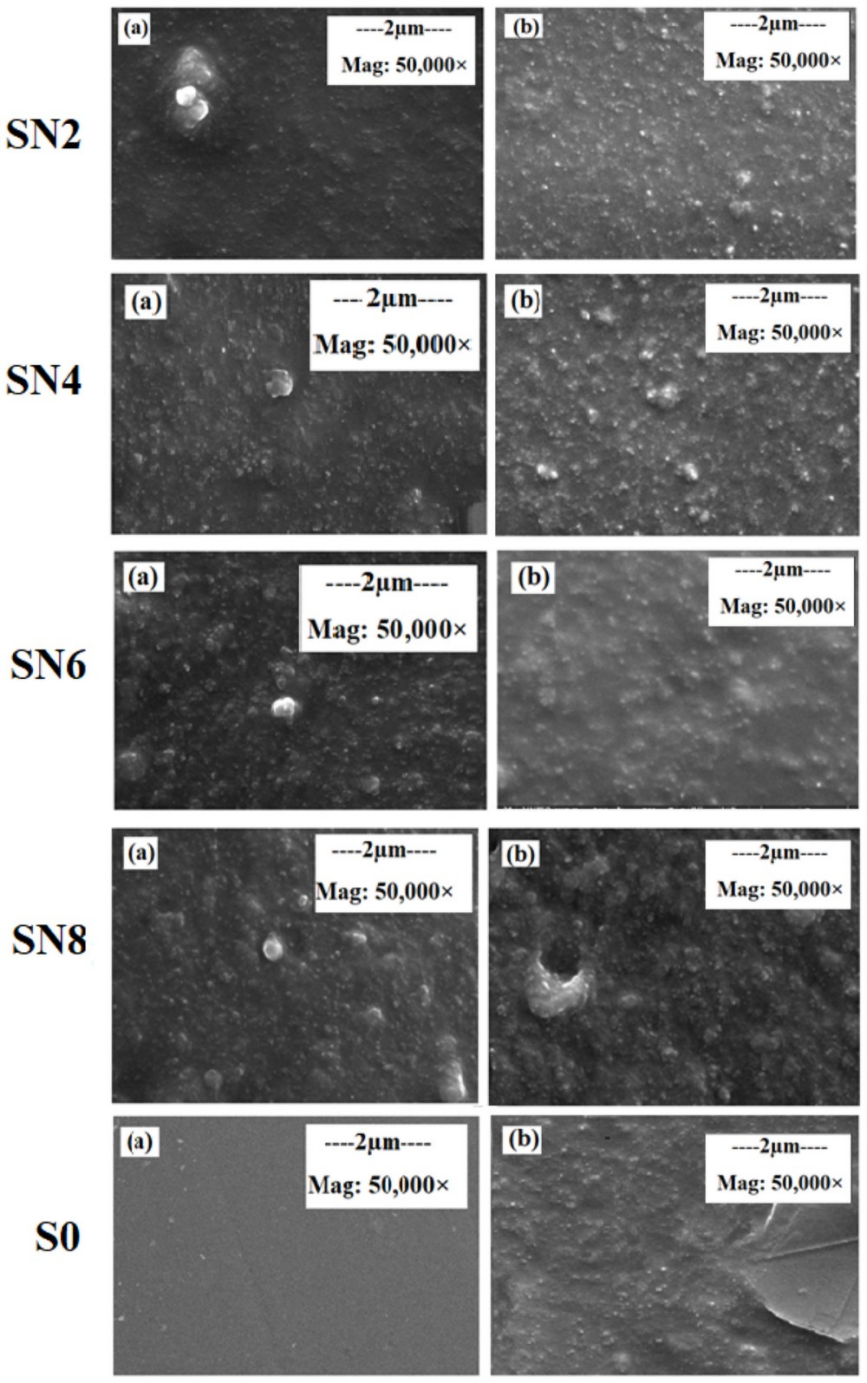
SEM a) Before aging, b) After aging of 9000 hours.

Scanning electron microscopy is performed to study the morphology of neat as well as hybrid samples. SEM analysis is conducted using field emission scanning electron microscope (FESEM, Hitachi U-5000, Japan). Fig 4 presents the SEM images for unaged and aged samples.

After aging, S0 has small cracks on the surface of the sample as compared to the hybrid samples. All the hybrid samples appeared to be less rough with less/no cracks on the surface. However, among all the hybrids, SN2 had higher surface intactness and less surface roughness as compared to the other hybrids. Therefore SN2 sample is less affected by the aging conditions. The main reason for better intactness of the surface in case of hybrid after 9000 hours of aging is due to the large surface area of nano-silica and the excessive presence of silonal group in case of the hybrids. So, the S0 sample without any nano-silica showed the highest degree of degradation when compared with the filled samples.

### 3.2 Hydrophobicity Classification (HC) analysis

**Fig 5:**
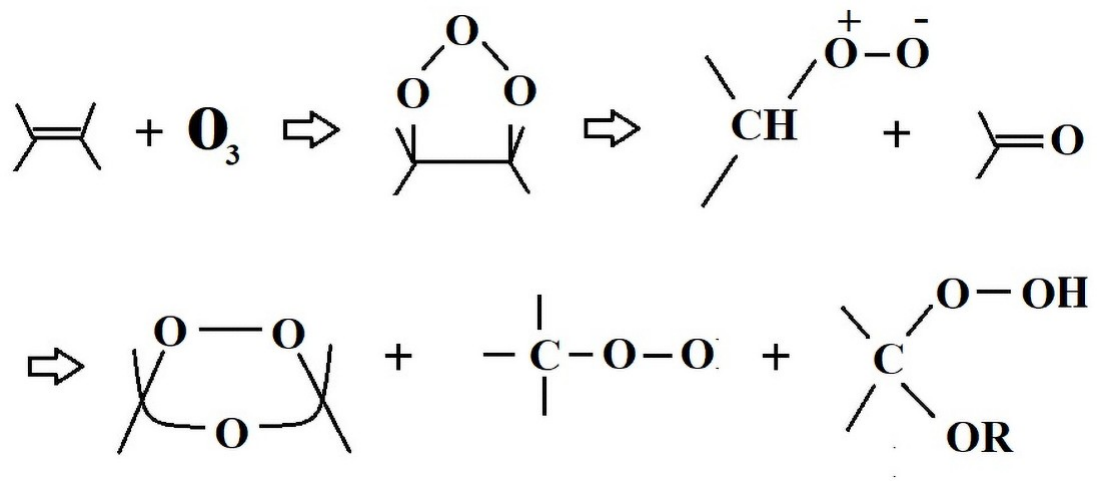
HC of samples for 9000 hours.

HC is performed by the method proposed by STRI (Sweden Transmission Research Institute) for outdoor insulators. Six hydrophobic class from HC1-HC6 are used, according to the pattern of the droplets sprayed on the surface of the insulators. HC4-HC6 represents the hydrophilic materials, indicating the warning sign. High pixel camera is used for taking the photographs and then compared with STRI guide photographs. Fig 5 shows the HC levels for all the samples after every cycle.

**Fig 6:**
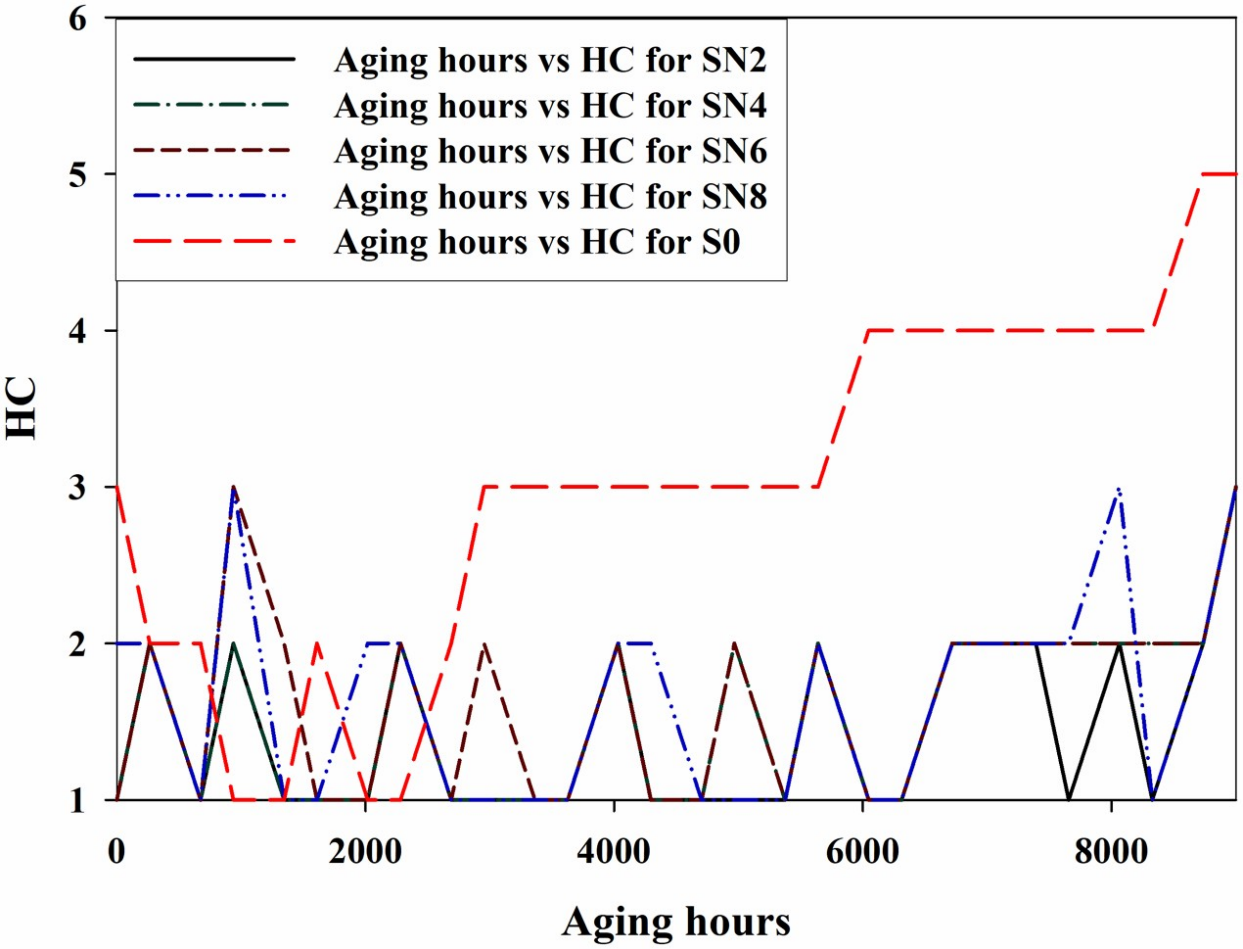
Silicone reaction mechanism with ozone.

**Fig 7:**
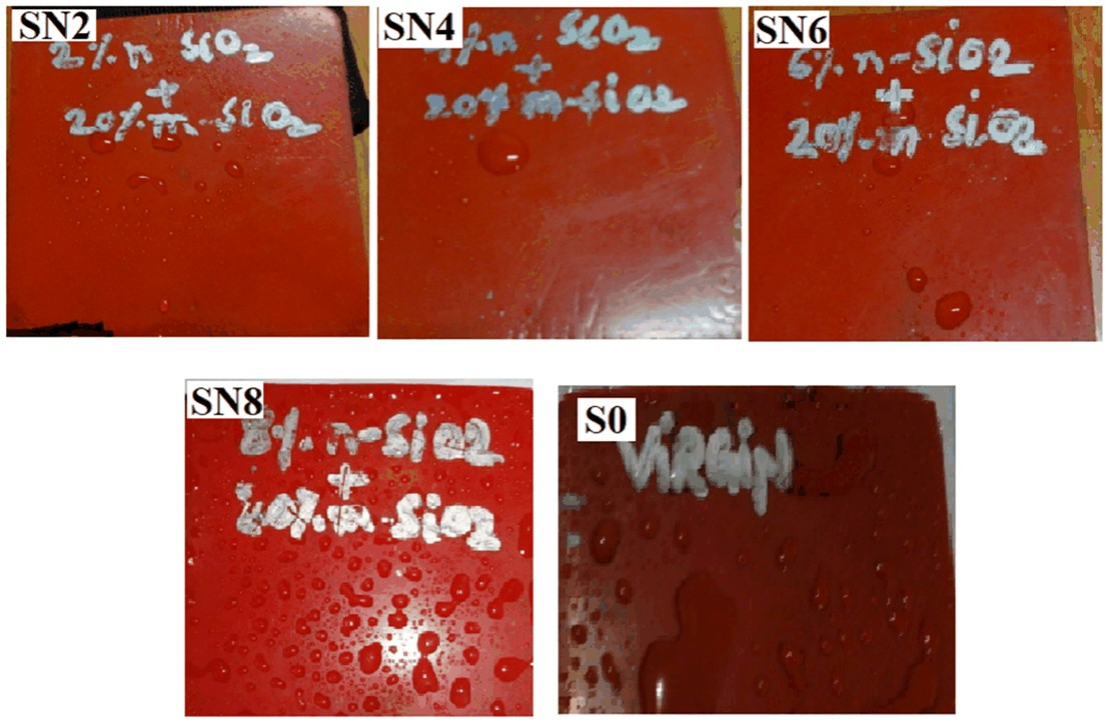
HC before aging.

**Fig 8:**
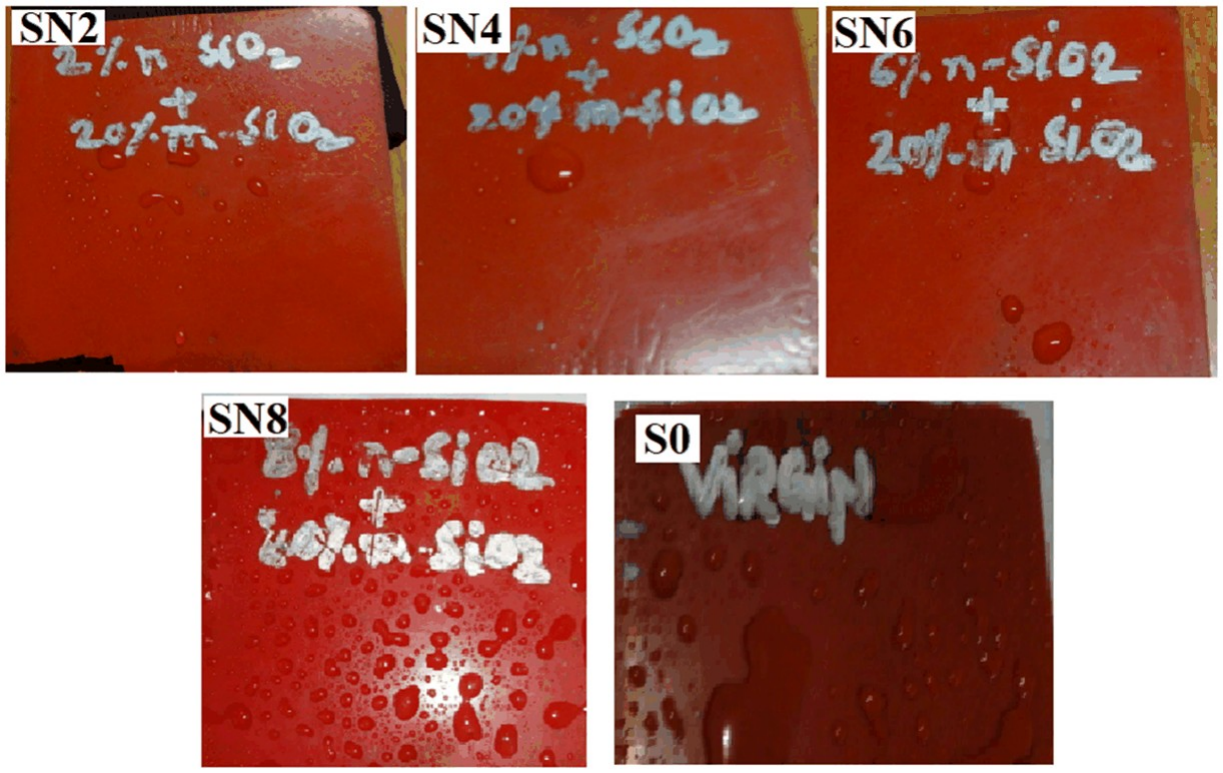
HC after 9000 hours of aging.

Before aging, the HC level by the SN2, SN4, and SN6 is HC1 whereas, SN8 obtained HC2 and S0 got the HC3. As the aging started, all the samples showed an increase in HC levels. Around 6000 hours, the samples started to have lower HC levels, thus indicating an improvement in the HC. SN2, SN4, and SN6 samples demonstrated the HC1 and HC2 level variation till 8736 hours. At the end of the aging, the highest HC level indicated by these three samples is HC3. SN8 showed HC3 around 8064 hours and ends with the same HC level. However, S0 sample started with HC3 level and at the end of 9000 hours of aging, it reaches to HC5 level, thus indicating the hydrophilic behavior of the sample. S0 also has the highest leakage current among all the samples. Hybrids i.e., SN2, SN4, and SN6 are the ones with improved HC levels as compared to the S0. The application of high voltage and harsh weather conditions (ultra violet radiations) on S0 causes the depolarization by producing ozone and ionic particles. The stresses in the environment affect the insulation chemically by changing the macromolecular chain to low weight molecules (LWMs). The deterioration of the insulation is caused by initiation of chain reaction forming stronger polar bonds, such as –OH, –C = O and C-O. These bonds start the molecular structure’s cross linking, tree and pore formation. The reaction of the mechanism is given in Fig 6. The high HC level is due to the microscopic conductive paths. In [23, 24], the loss of hydrophobicity is due to the algae contamination. However, the recovery in the HC level is because of the movement of lower molecular chains to the surface. Figs 7 and 8 show HC before and after aging results.

The recovery phenomenon is observed by all samples and it is the characteristic of a base material [23]. This recovery phenomenon is more pronounced in the hybrid samples as compared to S0. The main reason for the improved performance of hybrids (SN2, SN4, and SN6) is due to the better interface of micro and nano-silica with the base material. In case of SN8, the higher loading of the nano-particle gives the saturation effects and instead of increasing the filler and base material interaction, it simply decreases the filler and base interaction. Therefore, increasing the hydrophobicity level.

### 3.3 Leakage Current (LC)

**Fig 9:**
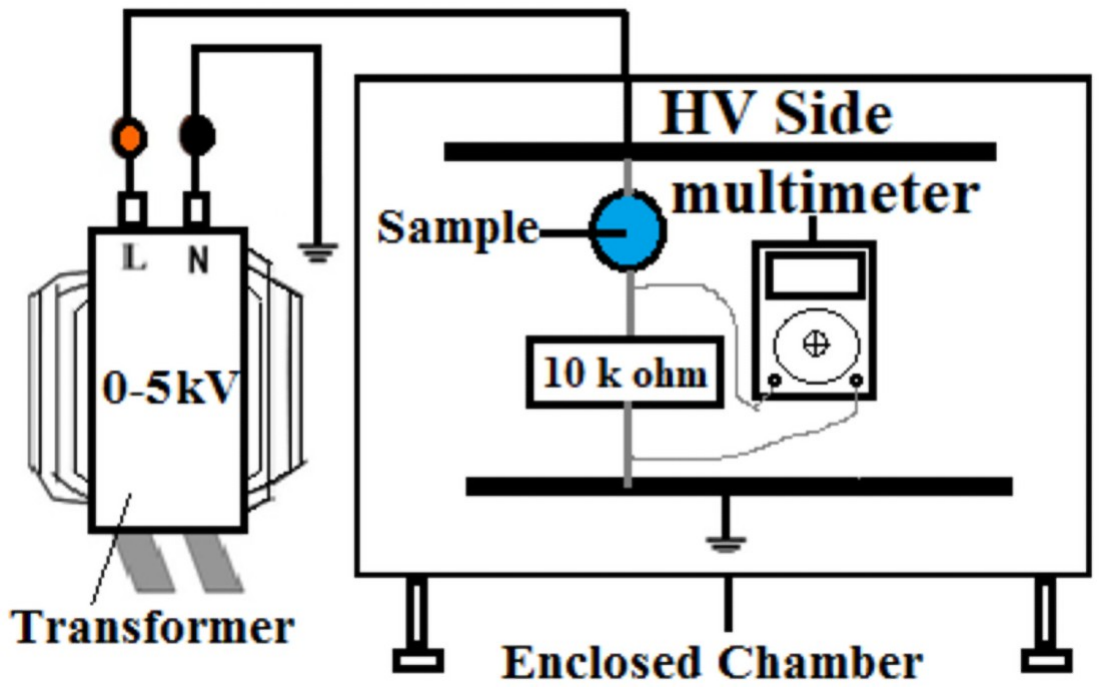
Leakage current setup.

LC is a promising parameter for accessing the condition of an insulator when placed in the field [25]. Closed chamber is utilized for measuring the LC. The enclosed chamber and setup is shown in Fig 9. A 10 kΩ resistor is placed in series with the polymer and a 2kV source voltage is applied. Voltage is measured across the sample with the help of a multimeter. Consecutive five values are measured to calculate an average value. LC value is obtained by using the Ohm’s law. The LC is measured for every winter and summer cycle for 9000 hours of artificial aging.

**Fig 10:**
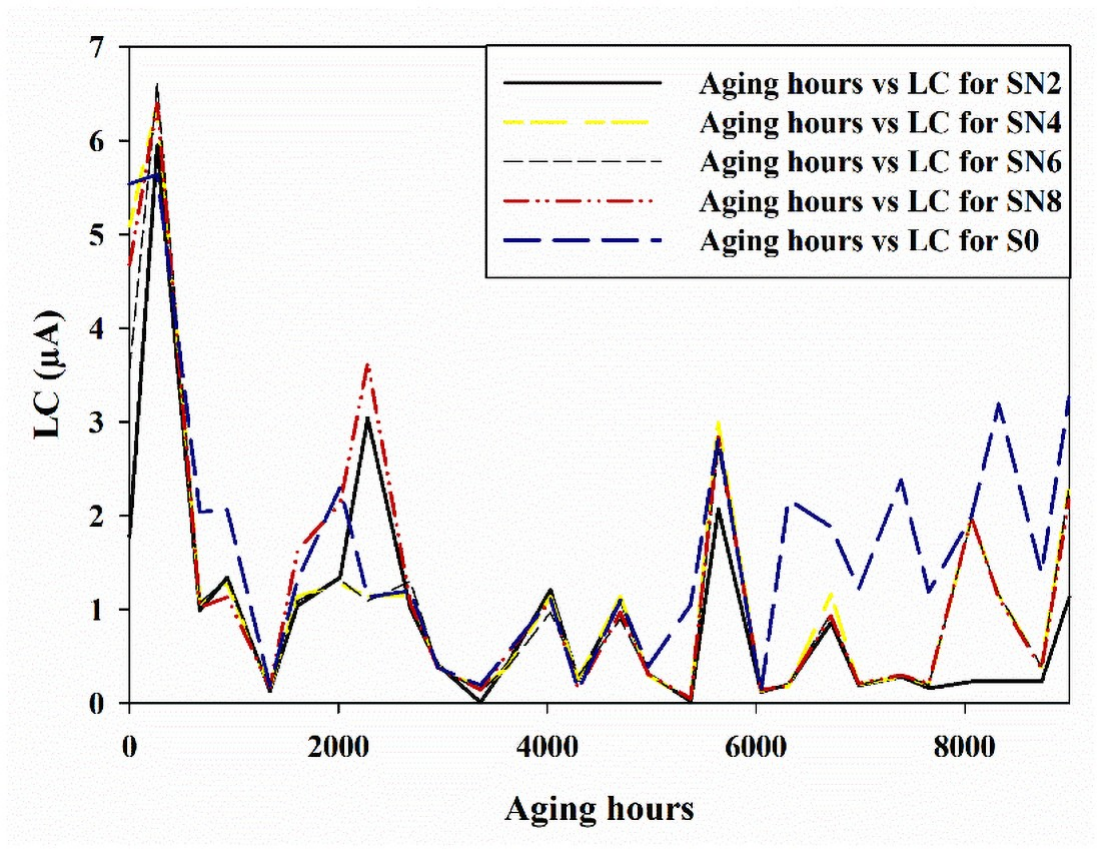
Leakage current aged from 0 hours-9000 hours.

The values are recorded at 40% humidity and 25°C as LC values are greatly affected by the variation in temperature and humidity [20]. The LC is presented in Fig 10 for every summer and winter cycle for the entire 9000 hours of aging.

Without stress application, the highest LC value of 5.54 *μ*A is calculated for S0 sample, whereas the lowest LC of 1.77 *μ*A is for the SN2 sample. As the aging started, an increase in the value of LC was observed for all the samples. The reason for this increase in LC is the relocation of the methyl group [16]. The highest value for the entire aging time of 9000 hours is measured at 264 hours of 6.6 *μ*A by the sample SN6. However, after the increase in LC, all the samples initiated to recover themselves. Our hybrid samples SN2, SN4, and SN6 have the lowest LC value as compared to the S0 sample throughout the artificial aging.

However, SN8 exhibited higher values as compared to the S0 sample. After 2000 hours of aging, again rise in LC is observed and then again recovery pattern is observed. Around 5500 hours, the rise in LC and after that recovery in the LC is measured. The recovery after 5500 hours is not as significant as it is after 2000 hours of aging. At the end of the aging, all the samples showed a trend in the increased values of LC values.

After aging, the highest LC of 3.27 *μ*A is exhibited by the S0 sample and the lowest LC of 1.77 *μ*A is exhibited by SN2 sample. Three hybrids SN2, SN4, and SN6 showed exclusively improved performance as compared to the S0 sample.

Overall, SN2 sample is the one with lowest LC values and thus less affected by the aging stresses. The main reason for increase in LC and less recovery is because of degradation in the polymer due to its exposure to the UV radiation. This phenomenon of the polymer degradation is also observed by other authors [26].

### 3.4 Breakdown strength

The breakdown strength is evaluated according to the standard ASTM D-149. The voltage rise of 200 V/s with a frequency of 50 Hz is used for this test. Dimensions are selected according to the section II and type II electrodes are used. Electrodes and sample are dipped in oil during the breakdown test.

**Fig 11:**
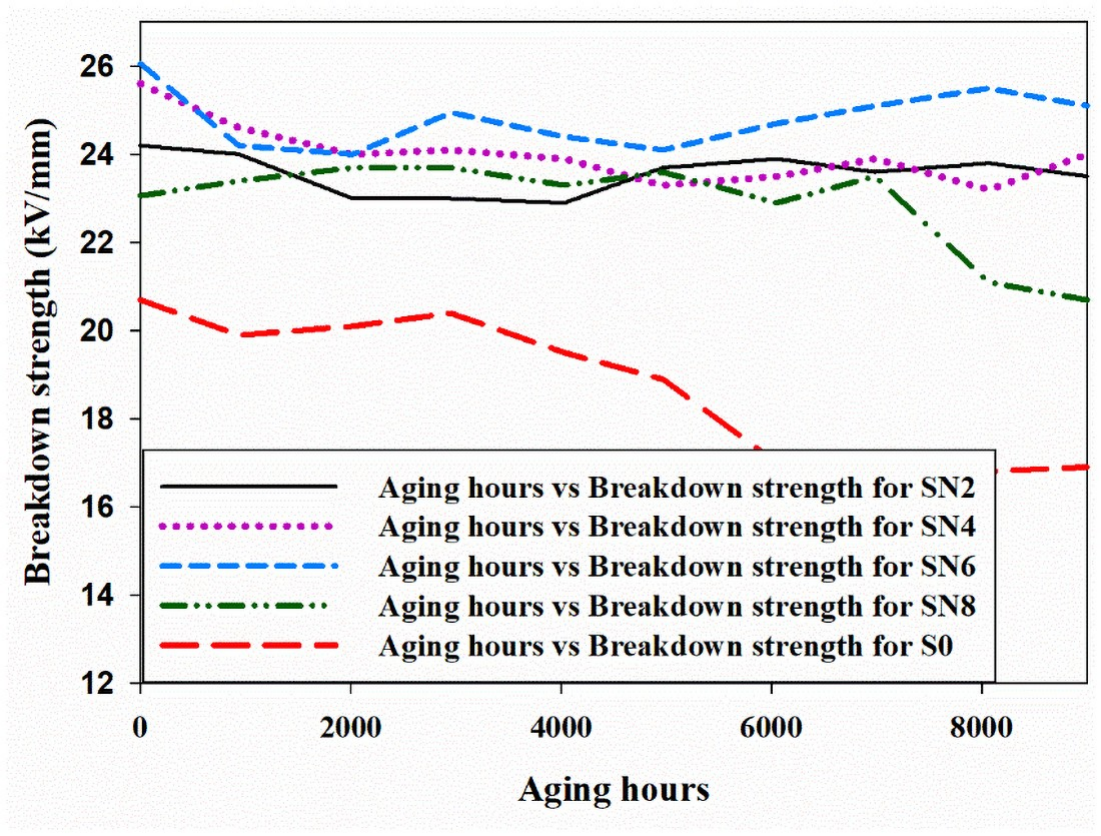
Breakdown strength.

From Fig 11, it can be deduced that the highest breakdown value is measured for the SN6 sample whereas the lowest value is observed for S0 sample. S0 samples have the lowest breakdown strength for the entire 9000 hours of artificial aging. Breakdown strength is reduced after aging for all the hybrids. Between 2000 hours to 4000 hours, a similar trend in the values are observed for all the samples. After that, loss and gain recovery pattern is noticed for all the samples. SN6 sample started recovering itself after 6000 hours, whereas SN2, SN4, and SN8 again loss breakdown value around 6000 hours of aging. The lowest breakdown strength is exhibited by S0, thus making it the worst option among all the samples after aging. All the hybrid samples responded significantly to the multi stresses, whereas S0 sample is highly affected by the applied stresses. Among all the hybrid samples, SN8 is the one with the lowest breakdown strength value of 23.06 kV/mm. Still this value is higher as compared to the virgin sample. Overall, the hybrids have superior performance in breakdown strength after 9000 hours of aging.

The improved result in case of hybrid is due to the better cross-linking between the nano and micro filler with the base material. This addition of nano and micro filler provides the higher bond energy, homogenous structure, and less surface energy, which results in improved breakdown strength. It was observed that the addition of nano and micro filler improves the breakdown strength [27]. Similar behavior in the case of hybrid is noticed in our research.

### 3.5 FTIR analysis

FTIR is performed for the entire 9000 hours of aging after every weather cycle for analyzing the condition of important peaks. Perkin-Elmer Spectrum 2000 FTIR spectrometer is used for FTIR. FTIR is performed immediately at the end of each weather cycle. All the important chemical groups along with their wavenumbers are illustrated in Table 3.

**Table 3:** Informations about wavelength and the basic functional groups.

Band Number	Wavenumber (cm-1)	Functional Group
1	2963–2850	C-H in CH_3_
2	1440–1400	CH_3_ in Si-CH_3_
3	1280–1240	Si-CH_3_
4	1130–1000	Si-O-Si
5	840–790	Si(CH_3_)_2_
6	870–850	Si-O in O-Si(CH_3_)_3_
7	700	Si- of Si-(CH_3_)_3_

**Fig 12:**
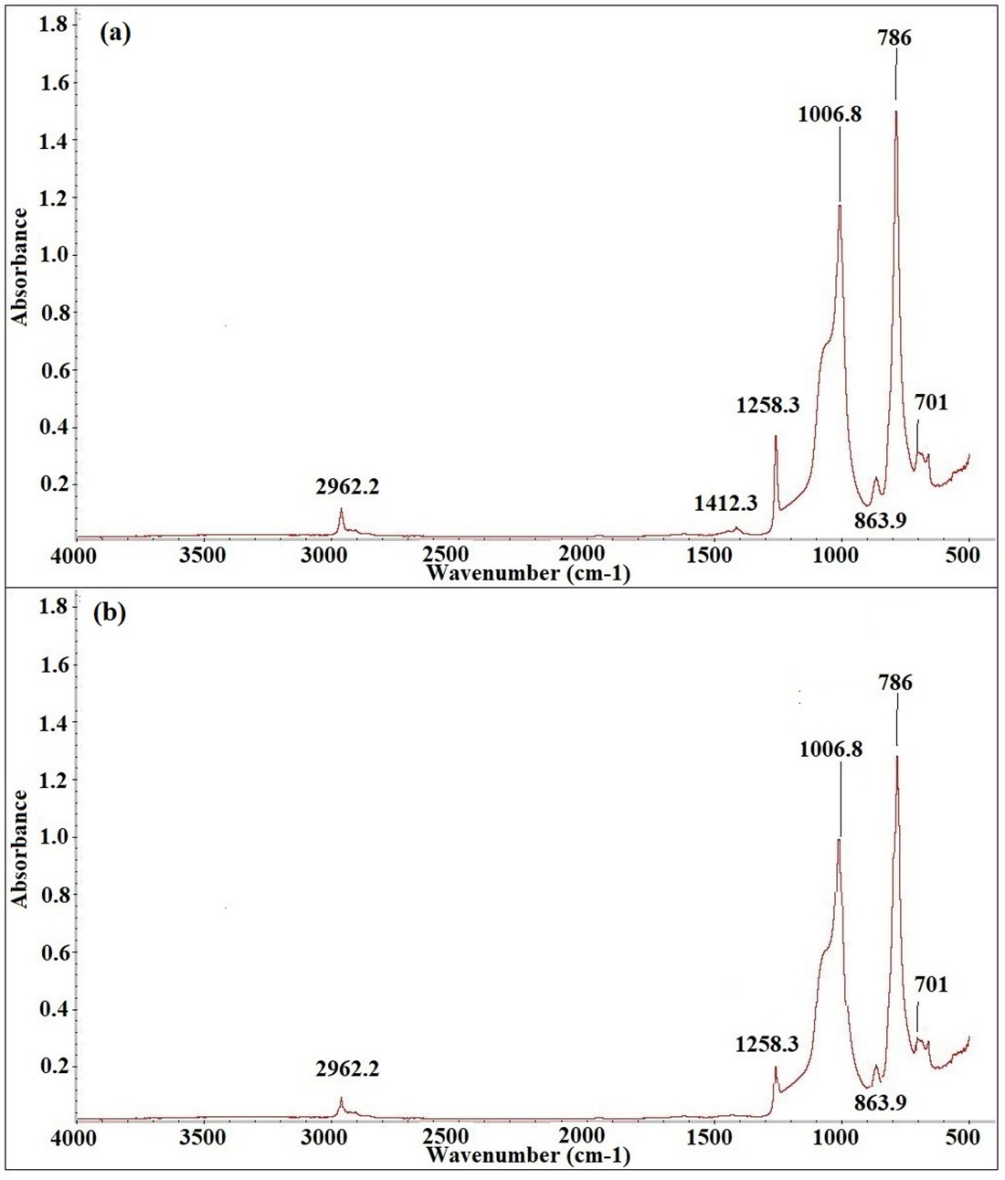
Spectrograph for SN2 a) Before aging, b) After aging.

**Fig 13:**
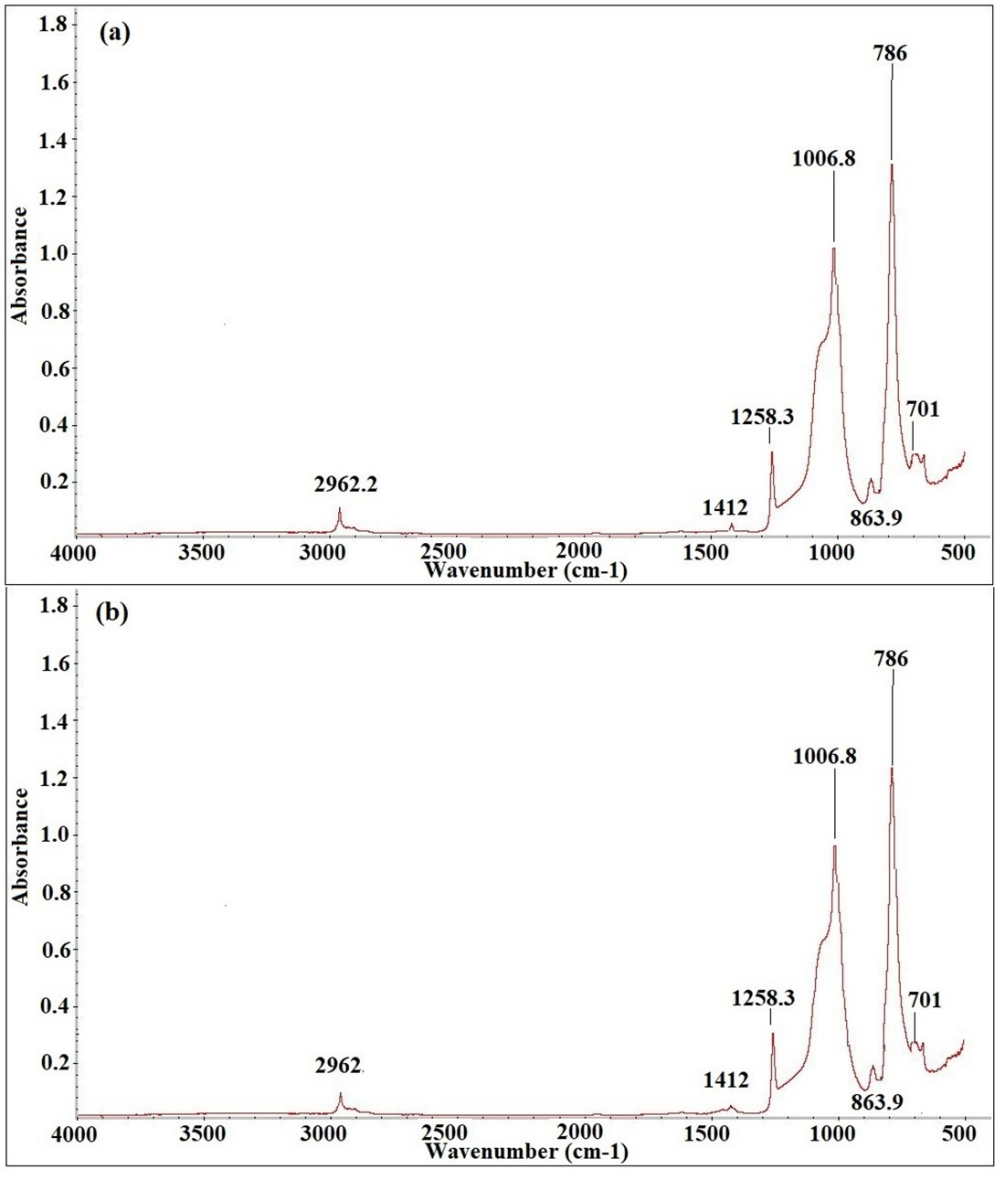
Spectrograph for SN4 a) Before aging, b) After aging.

**Fig 14:**
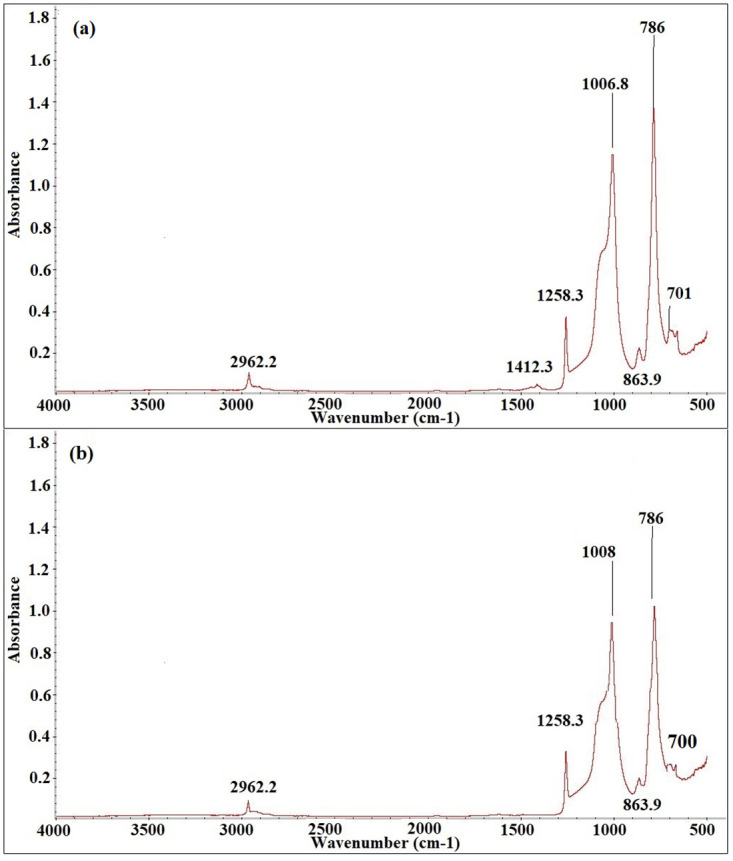
Spectrograph for SN6 a) Before aging, b) After aging.

**Fig 15:**
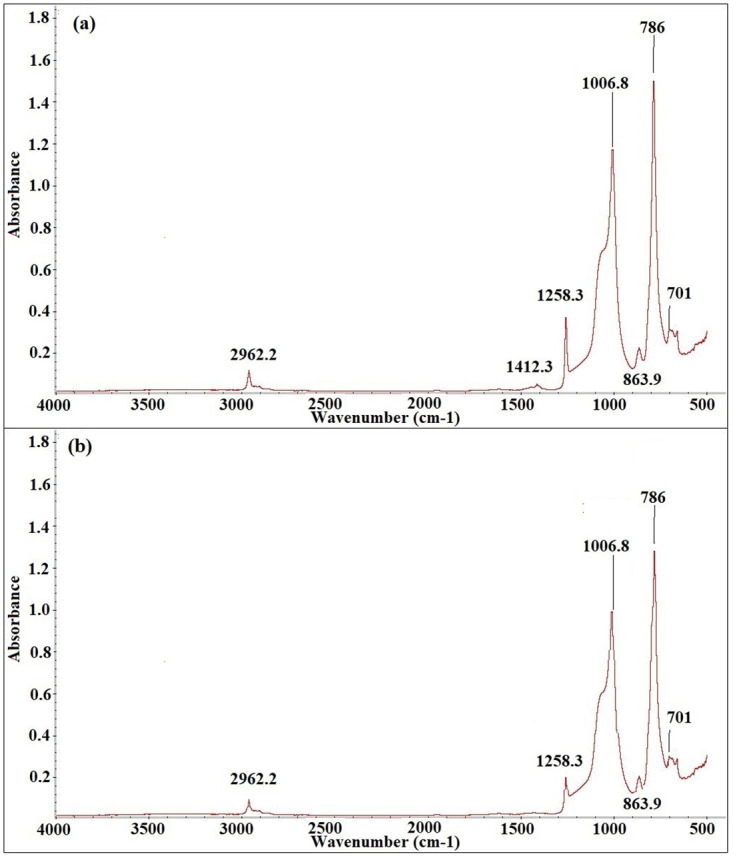
Spectrograph for SN8 a) Before aging, b) After aging.

**Fig 16:**
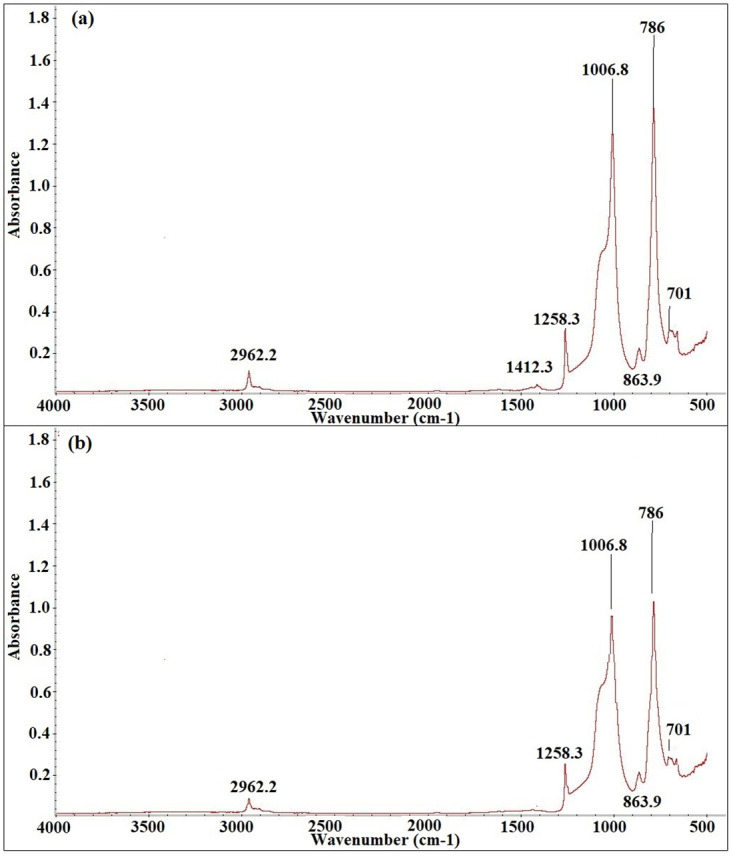
Spectrograph for S0 a) Before aging, b) After aging.

Figs 12–16 show before and after spectrographs for SN2, SN4, SN6, SN8, and S0. All the important intensities of the specific wavenumbers for all the samples are presented in the S1 File.

In Fig 12, the wavenumber and their respective absorbance peaks are shown for SN2. The certain absorbance peaks indicates the presence of important wavenumber numbers. The absorbance peaks are compared with the un-aged sample peaks throughout the aging time. Variation in the magnitude of the peaks show the degradation in the samples caused by the applied environmental stresses. From Fig 12, it can be observed that the wavenumber at 1440–1400 (cm-1) with bond CH_3_ in Si-CH_3_, is the only one that loses itself completely at the end of the aging. The wavenumbers at 2963–2850, 1280–1240, 1130–1000, 840–790, and 870–850 (cm-1) with bonds C-H in CH_3_, Si-CH_3_, Si-O-Si, Si(CH_3_)_2_, Si-O in O-Si(CH_3_)_3_, and Si- of Si-(CH_3_)_3_ lose 16.8%, 13.2%, 10.2%, 7.5%, and 5.14% at the end of 9000 hours of accelerated aging. A gain and loss pattern is found in all the samples. The loss pattern is found till 6000 hours, however the gain pattern is found around 7000 hours of aging. Then again a loss pattern is observed for all the samples. Wavenumber at 1440–1400 (cm-1) is the only wave which does not exhibit gain pattern at 7000 hours of aging.

In Fig 13, the spectrograph for SN4 with important wavenumber before and after aging can be observed. As the aging started, the sample started losing the intensity of certain peaks. At 936 hours, the percentage loss for wavenumbers 1258, 1008, 864, 786, and 700 (cm-1) for bond Si-CH_3_, Si-O-Si, Si(CH_3_)_2_, Si-O in O-Si(CH_3_)_3_, and Si- of Si-(CH_3_)_3_ is 1.62%, 3.15%, 11.37%, 0.8%, and 3.125%. Thus the aging started to show its effect on the structures of the important wavenumbers. Overall, loss and recovery pattern is found in this sample. Later, a gain pattern is found after 8000 hours of aging till the end. After 9000 hours of aging, the bonds C-H in CH_3_, Si-O-Si, Si(CH_3_)_2_, Si-O in O-Si(CH_3_)_3_, and Si- of Si-(CH_3_)_3_ have the percentage loss of 9%, 25%, 19%, 8, and 14%.

Fig 14 presents the variation pattern in wavenumber in spectrograph for SN6 before and after aging. Around 936 hours, the percentage loss by the bonds C-H in CH3, Si-CH_3_, Si-O-Si, Si(CH_3_)_2_, and Si-O in O-Si(CH_3_)_3_ are 13%, 19%, 16%, 21%, and 9.5%. The sample shows an increasing tendency in the loss till 6000 hours of aging. After 6000 hours of aging recovery pattern is observed. The highest loss observed for the bonds are 34%, 36%, 22.8%, 22.1, and 28%. At 9000 hours of aging, the percentage loss is 31%, 37.55, 20.1%, 21%, and 26% for the wavenumbers C-H in CH_3_, Si-(CH_3_)_2_, Si-O-Si, Si(CH_3_)_2_, and Si-O in O-Si(CH_3_)_3_.

For SN8, Fig 15 represents the FTIR analysis graph. As the aging starts, the percentage loss around 936 hours is highest among all the hybrid samples. The bonds C-H in CH_3_, Si-CH_3_, Si-O-Si, Si(CH_3_)_2_, Si-O in O-Si(CH_3_)_3_, and Si-Si(CH_3_)_3_ have the percentage loss of 16.66%, 25.6%, 48.16%, 33.33%, 32.66%, and 100%. The highest loss is observed around 6000 hours of aging for the same above bond number as 33%, 51.2%, 47.12%, 23.33%, and 100%. However, after 6100 hours of aging, the sample starts to recover itself and the percentage loss increases. Before 6000 hours of aging, the loss recovery pattern is observed but with small variation. However, the rise around 6000 hours of aging is significant. At the end of the aging, the samples have lower percentage loss for the bonds C-H in CH_3_, SiCH_3_, Si-O-Si, Si(CH_3_)_2_, Si-O in O-Si(CH_3_)_3_, and Si-Si(CH_3_)_3_ of 26.66%, 46.1%, 46%, 33%, 14%, and 33.33%.

Before and after aging spectrograph for S0 sample is presented in Fig 16. For S0 sample, the loss recovery pattern is observed by all the absorbance peaks. However, the percentage loss for some of the peaks mentioned in Table 4 is highest as compared to the hybrid samples. CH_3_ bond in Si-CH_3_ at the wavenumber 1440–1400 (cm-1), loses itself 100% and is unable to recover. Wavenumbers 1130–1000 (cm-1) for Si-O-Si bond, 840–790 (cm-1) for Si-(CH_3_)_2_, and 870–850 (cm-1) for Si-O bond in O-Si(CH_3_)_3_ are the ones with percentage loss of 25% at the end of the experiment. Loss recovery pattern is observed for all the wavenumbers except 1440–1400 (cm-1). However, as compared to the other samples, this sample does not recover itself as much as the other hybrid samples do. It means this sample is highly affected by the weathering stresses applied on the samples. From The FTIR analysis for all the samples, we can conclude that the hybrid samples specially SN2 and SN4 are the ones with the lowest percentage loss as compared to the other hybrid and S0 sample. However, SN6 and SN8 recover themselves after having the highest loss percentage. In case of the S0 sample, loss recovery pattern exists but the recovery is not significant as compared to the hybrid samples. The groups Si-CH_3_, Si-O-Si, and C-H groups of silicone base material with a decrease in the absorbance peaks at significant wavenumber was also reported by other authors after aging [28, 29]. Loss and recovery is also observed by other authors for silicone rubber as base material [16]. The addition of the filler in the base material simply keeps it intact due to the addition of silanol group present on the nano filler, thus making it resistant to the aging conditions [30]. In our case, the addition of nano and micro particle enhances the recovery pattern of the hybrid samples when compared with the unfilled sample after weathering effects.

**Table 4:** Comparison with state-of-art work, here ′-′ indicates the gain.

References	No. Of Samples	Time Period/Type for Aging	Composition Of Samples	Wavenumbers (cm-1)	Absorbance, peak percentage loss at the end of aging	Breakdown strength in percentage loss after aging	Hydrophobicity	Leakage current in percentage loss after aging (*μ*A)
Our Research Work	SN2		2% nano SiO_2_+20% micro SiO_2_% in HTV-SiR%	2963–2850	16.82	2.9	HC1-HC3	36
				1440–1400	100			
				1280–1240	13.24			
				1130–1000	10.23			
				840–790	7.5			
				870–850	5.14			
				700	3.34			
	SN4	9000 hours/Artificial	4% nano SiO_2_+20% micro SiO_2_ in HTV-SiR	2963–2850	8.75	6.7	HC1-HC3	54
				1440–1400	100			
				1280–1240	24.19			
				1130–1000	18.9			
				840–790	19.54			
				870–850	7.69			
				700	14.06			
	SN6		6% nano SiO_2_+20% micro SiO_2_ in HTV-SiR	2963-2850	34.78	5.5	HC1-HC3	36
				1440–1400	100			
				1280–1240	36.11			
				1130–1000	21.84			
				840–790	22.84			
				870–850	7.53			
				700	0			
	SN8		8% nano SiO_2_+20% micro SiO_2_ in HTV-SiR	2963-2850	26.66	17.5	HC1-HC3	53
				1440–1400	100			
				1280–1240	46.15			
				1130–1000	46.07			
				840–790	33.33			
				870–850	14.66			
				700	33.33			
	S0		Only HTV-SiR (neat)	2963–2850	31.57	23	HC3-HC5	40.9
				1440–1400	100			
				1280–1240	32.43			
				1130–1000	25			
				840–790	25			
				870–850	25			
				700	0			
[31]	SiR-2	9000 hours	10% micro silica and 5% Nano silica	2963–2960	-20	−	HC1-HC2	100
				1280–1225	3.1			
				1110–1050	11			
				1130–1000	22			
				870–850	21			
				840–790	28			
				700	24			
[32]	one Sample	5000 hours	Silicone rubber with a filler	5000 hours	−	23.8	−	23.7
[33]	391 Samples	3–22 years	Silicone Rubber+ATH	3400 3600	40.9	−	−	−
				1280 1255	97.8			
				1100 1050	88			
				1130 1000	95.89			
				840 890	85			
[34]	SR	7 Days	Unfilled HTV Silicone Rubber	786	68.6	−	−	−
				1010	41.9			
				1258	52			
				2962	32			
				2962	32			
	MSR		Only 40% micro ATH	786	67.7			
				1010	43			
				1258	44.89			
				2962	14			
	MSR1		1% nano Alumina and 39% micro ATH	786	55.9			
				1010	28			
				1258	47			
				2962	31.2			
	MSR2		2% Nano Alumina and 38% micro ATH	786	58.9			
				1010	32			
				1258	46			
				2962	25			

## 4 Comparative analysis and discussion with the state-of-art works

In Table 4, our research work is critically compared with the recently published state-of-art research in terms of long term aging characteristics of insulator materials. In [31], the authors investigated the long term aging of silicon rubber with 10% micro silica and 5% micro silica for 9000 hours. The overlapping wavenumbers show comparable loss percentages when compared with our SN2 sample, however HC levels were same as our SN2. In [32], the insulators are aged for 5000 hours after that the breakdown strength and leakage current are measured. The results indicate more loss in breakdown strength when compared to our SN2 sample. However, in case of the leakage current, our sample had higher loss. The reason is due to the extended aging time period that makes the surface more rough, thus having high leakage current value. In [33], the researchers investigated the real time aging characteristics for 22 years in terms of FTIR analysis. As the samples were aged for a longer period, the results indicated higher deterioration for the specific wavenumbers. In research presented in [34], the hybrid samples were prepared and placed in a chamber for extreme corona for seven days. For aging characteristics analysis FTIR was performed. The results indicated that their sample MSR1(39% micro ATH+1% nano alumina) exhibited the minimum loss percentage for specific wavenumber. Comparing the work in [34] with this research, our SN2 sample presents the lowest percentage loss after 9000 aging hours. Therefore concluding that our sample SN2 has the improved aging characteristics when compared with other state-of-art research work done so far, making it a good option for selection. The reason for an improved characteristics in case of SN2 is the unique nano and micro-silica percentages and superior interaction of these particles with the base material. Thus, SN2 possessed improved LC, HC, breakdown strength and aging characteristics.

## 5 Conclusion

This research paper represents the aging phenomenon of the nano-silica and micro-silica in HTV-SiR hybrids composite insulators. Neat and hybrid samples are prepared and then subjected to long term accelerated aging of 9000 hours for further investigation. Hybrid samples SN2, SN4, and SN6 shows improved aging characteristics whereas SN8 sample shows adverse behavior when compared with S0. The SEM analysis shows the formation of cracks for S0 samples but the simultaneous addition of nano and micro-silica prevented the formation of the cracks. SN2, SN4, and SN6 has the varying hydrophobicity classification of HC1-HC2, whereas SN8 and neat samples have higher HC levels. SN2 does not lose significant breakdown strength even after aging of 9000 hours. Leakage current variation throughout the 9000 hours is found to be lowest for SN2. In case of FTIR analysis, the percentage loss for SN2 is lowest among all the hybrid samples and neat sample. Overall, all the hybrids performed well except SN8. Addition of nano-silica more than 4% does not contribute to the improved aging characteristics. Therefore, SN2 and SN4 are the ones with superior aging characteristics for the application of high voltage insulators. In future, the influence of varying loadings of nano-silica with different filler sizes and percentages in micro range needs to be further investigated for different environmental conditions.

## Supporting information

S1 File (DOCX)
