# Supplementary material for: Investigation of 9000 hours multi-stress aging effects on High-Temperature Vulcanized Silicone Rubber with silica (nano/micro) filler hybrid composite insulator
Source: PLoS One. 2021 Jul 28;16(7):e0253372. doi: 10.1371/journal.pone.0253372 (PMC8318273; doi:10.1371/journal.pone.0253372)
Supplement: S1 File — (DOCX) [file pone.0253372.s001.docx]

Hydrophobicity Classification (Figure 5)

| Aging Time/Samples | SN2 | SN4 | SN6 | SN8 | S0 |
| --- | --- | --- | --- | --- | --- |
| 0 | HC1 | HC1 | HC1 | HC2 | HC3 |
| 264 | HC2 | HC2 | HC2 | HC2 | HC2 |
| 672 | HC1 | HC1 | HC1 | HC1 | HC2 |
| 936 | HC2 | HC2 | HC3 | HC3 | HC1 |
| 1344 | HC1 | HC1 | HC2 | HC1 | HC1 |
| 1608 | HC1 | HC1 | HC1 | HC1 | HC2 |
| 2016 | HC1 | HC1 | HC1 | HC2 | HC1 |
| 2280 | HC2 | HC2 | HC2 | HC2 | HC1 |
| 2688 | HC1 | HC1 | HC1 | HC1 | HC2 |
| 2952 | HC1 | HC1 | HC2 | HC1 | HC3 |
| 3360 | HC1 | HC1 | HC1 | HC1 | HC3 |
| 3624 | HC1 | HC1 | HC1 | HC1 | HC3 |
| 4032 | HC2 | HC2 | HC2 | HC2 | HC2 |
| 4296 | HC1 | HC1 | HC1 | HC2 | HC3 |
| 4704 | HC1 | HC1 | HC1 | HC1 | HC3 |
| 4968 | HC 1 | HC 2 | HC2 | HC1 | HC3 |
| 5376 | HC 1 | HC 1 | HC1 | HC1 | HC3 |
| 5640 | HC 2 | HC 2 | HC2 | HC2 | HC3 |
| 6048 | HC 1 | HC 1 | HC1 | HC1 | HC4 |
| 6312 | HC 1 | HC 1 | HC1 | HC1 | HC4 |
| 6720 | HC 2 | HC 2 | HC2 | HC2 | HC4 |
| 6984 | HC 2 | HC 2 | HC2 | HC2 | HC4 |
| 7392 | HC 2 | HC 2 | HC2 | HC2 | HC4 |
| 7656 | HC 1 | HC 2 | HC2 | HC2 | HC4 |
| 8064 | HC 2 | HC 2 | HC2 | HC3 | HC4 |
| 8328 | HC 1 | HC 2 | HC2 | HC1 | HC4 |
| 8736 | HC 2 | HC 2 | HC2 | HC2 | HC5 |
| 9000 | HC 3 | HC 3 | HC3 | HC3 | HC5 |

Leakage current (Figure 10)

| Hours | SN2 | SN4 | SN6 | SN8 | S0 |
| --- | --- | --- | --- | --- | --- |
| 0 | 1.77 | 5.09 | 3.56 | 4.68 | 5.54 |
| 264 | 5.25 | 6.4 | 6.6 | 6.4 | 5.64 |
| 672 | 0.99 | 1.07 | 1.07 | 1.02 | 2.04 |
| 936 | 1.34 | 1.27 | 1.32 | 1.13 | 2.06 |
| 1344 | 0.13 | 0.16 | 0.16 | 0.17 | 0.18 |
| 1608 | 1.04 | 1.14 | 1.09 | 1.63 | 1.32 |
| 2016 | 1.34 | 1.27 | 1.32 | 2.13 | 2.3 |
| 2280 | 3.04 | 1.14 | 1.09 | 3.63 | 1.13 |
| 2688 | 1.01 | 1.15 | 1.3 | 1.07 | 1.2 |
| 2952 | 0.41 | 0.39 | 0.39 | 0.38 | 0.38 |
| 3360 | 0.01 | 0.15 | 0.13 | 0.14 | 0.19 |
| 3624 | 0.43 | 0.39 | 0.39 | 0.54 | 0.55 |
| 4032 | 1.21 | 1.14 | 0.97 | 1.1 | 1.11 |
| 4296 | 0.25 | 0.26 | 0.29 | 0.15 | 0.14 |
| 4704 | 1.1 | 1.14 | 0.9 | 0.97 | 1.1 |
| 4968 | 0.31 | 0.3 | 0.3 | 0.31 | 0.39 |
| 5376 | 0.02 | 0.03 | 0.02 | 0.05 | 1.05 |
| 5640 | 2.07 | 2.99 | 2.73 | 2.84 | 2.83 |
| 6048 | 0.12 | 0.13 | 0.13 | 0.14 | 0.12 |
| 6312 | 0.2 | 0.18 | 0.2 | 0.19 | 2.17 |
| 6720 | 0.86 | 1.16 | 0.96 | 0.93 | 1.88 |
| 6984 | 0.19 | 0.2 | 0.19 | 0.21 | 1.22 |
| 7392 | 0.28 | 0.28 | 0.31 | 0.3 | 2.38 |
| 7656 | 0.16 | 0.2 | 0.19 | 0.21 | 1.19 |
| 8064 | 0.23 | 1.97 | 1.96 | 1.98 | 1.99 |
| 8328 | 0.23 | 1.15 | 1.16 | 1.12 | 3.19 |
| 8736 | 0.23 | 0.39 | 0.39 | 0.37 | 1.39 |
| 9000 | 1.77 | 2.33 | 2.3 | 2.17 | 3.27 |

Breakdown strength (Figure 11)

| Hours | SN2 | SN4 | SN6 | SN8 | S0 |
| --- | --- | --- | --- | --- | --- |
| 0 | 24.2 | 25.6 | 26.6 | 23.06 | 21 |
| 1000 | 23.2 | 24.8 | 24.6 | 23.4 | 20 |
| 2000 | 22.8 | 24.2 | 24.6 | 23.7 | 20.1 |
| 3000 | 23 | 24 | 25.4 | 23.8 | 20.9 |
| 4000 | 22.9 | 24 | 25.3 | 23.2 | 19.4 |
| 5000 | 23.7 | 23.9 | 25.1 | 24.2 | 18.6 |
| 6000 | 23.9 | 24.1 | 25.7 | 22.9 | 16.5 |
| 7000 | 23.6 | 24.5 | 25.8 | 23.9 | 16.4 |
| 8000 | 23.8 | 23.1 | 25.9 | 21.7 | 16.7 |
| 9000 | 23.3 | 24 | 25.1 | 20.7 | 16.9 |

FTIR

Intensities of major absorption peaks in SN2 before and after aging (Figure 12)

| Wavenumber/Hours | 2963-2850 (C-H stretching in CH_3_) | 1440-1410  (CH_3_ in Si-CH_3_) | 1280-1250  (Si–CH_3_) | 1130-1000  (Si-O-Si stretching) | 870-850  (Si-O of O-Si(CH_3_)_3_) | 840-780  (Si-O of O-Si(CH_3_)_2_-O) | 700-600  (Si- of Si-(CH_3_)_3_) |
| --- | --- | --- | --- | --- | --- | --- | --- |
| 0 | 0.095 | 0.03 | 0.37 | 1.28 | 0.2 | 1.36 | 0.299 |
| 9000 | 0.079 | 0 | 0.321 | 1.149 | 0.185 | 1.29 | 0.289 |

Intensities of major absorption peaks in SN4 before and after aging (Figure 13)

| Wavenumbers/Hours | 2963-2850 (C-H stretching in CH_3_) | 1440-1410  (CH_3_ in Si-CH_3_) | 1280-1250  (Si–CH_3_) | 1130-1000  (Si-O-Si stretching) | 870-850  (Si-O of O-Si(CH_3_)_3_) | 840-780  (Si-O of O-Si(CH_3_)_2_-O) | 700-600  (Si- of Si-(CH_3_)_3_) |
| --- | --- | --- | --- | --- | --- | --- | --- |
| 0 | 0.08 | 0 | 0.31 | 0.95 | 0.22 | 1.3 | 0.32 |
| 9000 | 0.073 | 0.03 | 0.235 | 0.77 | 0.177 | 1.2 | 0.275 |

Intensities of major absorption peaks in SN6 before and after aging (Figure 14)

| Wave numbers/ Hours | 2963-2850 (C-H stretching in CH_3_) | 1440-1410  (CH_3_ in Si-CH_3_) | 1280-1250  (Si–CH_3_) | 1130-1000  (Si-O-Si stretching) | 870-850  (Si-O of O-Si(CH_3_)_3_) | 840-780  (Si-O of O-Si(CH_3_)_2_-O) | 700-600  (Si- of Si-(CH_3_)_3_) |
| --- | --- | --- | --- | --- | --- | --- | --- |
| 0 | 0.092 | 0 | 0.36 | 1.19 | 0.23 | 1.38 | 0.29 |
| 9000 | 0.062 | 0 | 0.225 | 0.95 | 0.18 | 1.02 | 0.21 |

Intensities of major absorption peaks in SN8 before and after aging (Figure 15)

| Wave numbers/ Hours | 2963-2850 (C-H stretching in CH_3_) | 1440-1410  (CH_3_ in Si-CH_3_) | 1280-1250  (Si–CH_3_) | 1130-1000  (Si-O-Si stretching) | 870-850  (Si-O of O-Si(CH_3_)_3_) | 840-780  (Si-O of O-Si(CH_3_)_2_-O) | 700-600  (Si- of Si-(CH_3_)_3_) |
| --- | --- | --- | --- | --- | --- | --- | --- |
| 0 | 0.09 | 0.02 | 0.39 | 1.91 | 0.3 | 1.5 | 0.3 |
| 9000 | 0.066 | 0.03 | 0.21 | 1.03 | 0.2 | 1.28 | 0.2 |

Intensities of major absorption peaks in S0 before and after aging (Figure 16)

| Wavenumbers/Hours | 2963-2850 (C-H stretching in CH_3_) | 1440-1410  (CH_3_ in Si-CH_3_) | 1280-1250  (Si–CH_3_) | 1130-1000  (Si-O-Si stretching) | 870-850  (Si-O of O-Si(CH_3_)_3_) | 840-780  (Si-O of O-Si(CH_3_)_2_-O) | 700-600  (Si- of Si-(CH_3_)_3_) |
| --- | --- | --- | --- | --- | --- | --- | --- |
| 0 | 0.095 | 0.03 | 0.37 | 1.28 | 0.2 | 1.36 | 0.33 |
| 9000 | 0.065 | 0 | 0.25 | 0.96 | 0.15 | 1.02 | 0.21 |
